# Genome sequence of *Phaeobacter caeruleus* type strain (DSM 24564^T^), a surface-associated member of the marine *Roseobacter* clade

**DOI:** 10.4056/sigs.3927623

**Published:** 2013-07-30

**Authors:** Paul G. Beyersmann, Olga Chertkov, Jörn Petersen, Anne Fiebig, Amy Chen, Amrita Pati, Natalia Ivanova, Alla Lapidus, Lynne A. Goodwin, Patrick Chain, John C. Detter, Manfred Rohde, Sabine Gronow, Nikos C. Kyrpides, Tanja Woyke, Meinhard Simon, Markus Göker, Hans-Peter Klenk, Thorsten Brinkhoff

**Affiliations:** 1 Institute for Chemistry and Biology of the Marine Environment (ICMB), Oldenburg, Germany; 2 Los Alamos National Laboratory, Bioscience Division, Los Alamos, New Mexico, USA; 3 Leibniz Institute DSMZ - German Collection of Microorganisms and Cell Cultures, Braunschweig, Germany; 4 Biological Data Management and Technology Center, Lawrence Berkeley National Laboratory, Berkeley, California, USA; 5 DOE Joint Genome Institute, Walnut Creek, California, USA; 6 HZI – Helmholtz Centre for Infection Research, Braunschweig, Germany

**Keywords:** biofilm, motile, indigoidine, quorum sensing, siderophores, *Rhodobacteraceae*, *Alphaproteobacteria*

## Abstract

In 2009 *Phaeobacter caeruleus* was described as a novel species affiliated with the marine *Roseobacter* clade, which, in turn, belongs to the class *Alphaproteobacteria*. The genus *Phaeobacter* is well known for members that produce various secondary metabolites. Here we report of putative quorum sensing systems, based on the finding of six *N*-acyl-homoserine lactone synthetases, and show that the blue color of *P. caeruleus* is probably due to the production of the secondary metabolite indigoidine. Therefore, *P. caeruleus* might have inhibitory effects on other bacteria. In this study the genome of the type strain DSM 24564^T^ was sequenced, annotated and characterized. The 5,344,419 bp long genome with its seven plasmids contains 5,227 protein-coding genes (3,904 with a predicted function) and 108 RNA genes.

## Introduction

*Phaeobacter caeruleus* 13^T^ (= DSM 24564 = LMG 24369 = CCUG 55859) was isolated at the ISMAR-CNR Marine Station, Genoa, Italy, during an analysis of the microbial diversity of a marine electroactive biofilm from a tank of about 100 L seawater [1]. The biofilm was grown on a cathodically polarized stainless-steel cathode [2]. In addition to *P. caeruleus* the genus consists of four other species, *P. arcticus*, *P. daeponensis*, *P. gallaeciensis* and *P. inhibens* and belongs to the *Roseobacter* clade, one of the most intensively studied groups of marine bacteria in recent years [3]. The clade belongs to the family *Rhodobacteraceae* within the class *Alphaproteobacteria*. *P. caeruleus* is named after the colony color of the isolates (cae.ru’le.us; L. masc. adj. *caeruleus* = dark-blue colored) [1]. Since the first publication, no further research on *P. caeruleus* was published. Therefore, we present for the first time a description and analysis of the high-quality draft genome sequence and annotation, including insights on genes coding for putative secondary metabolites like the blue pigment indigoidine or the quorum sensing mediating *N*-acyl-homoserine lactones. Furthermore, we summarize features of the organism, including novel aspects of its phenotype.

## Classification and features

### 16S rRNA gene analysis

**Figure 1:**
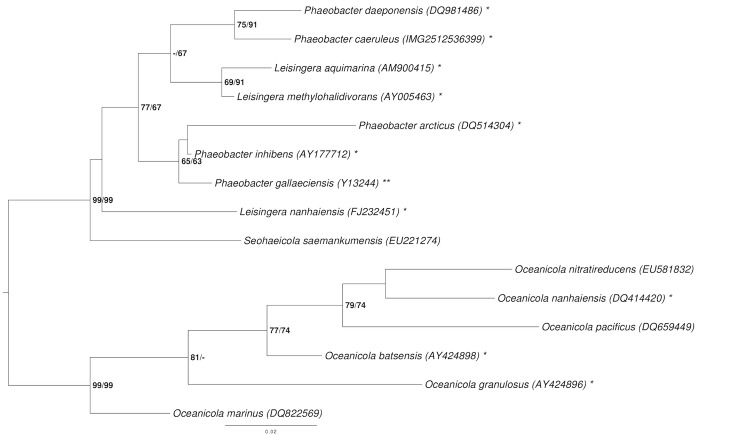
Phylogenetic tree highlighting the position of *P. caeruleus* relative to the type strains of the other species within the genus *Phaeobacter* and the neighboring genera *Leisingera* and *Oceanicola* [4-17]. The tree was inferred from 1,387 aligned characters [18,19] of the 16S rRNA gene sequence under the maximum likelihood (ML) criterion [20]. *Oceanicola* spp. were included in the dataset for use as outgroup taxa. The branches are scaled in terms of the expected number of substitutions per site. Numbers adjacent to the branches are support values from 1,000 ML bootstrap replicates [21] (left) and from 1,000 maximum-parsimony bootstrap replicates [22] (right) if larger than 60%. Lineages with type strain genome sequencing projects registered in GOLD [23] are labeled with one asterisk, those also listed as 'Complete and Published' with two asterisks [24]. New genome sequences are reported in this issue [9].

Figure 1 shows the phylogenetic neighborhood of *P. caeruleus* in a tree based on 16S rRNA gene sequences. The sequences of the four 16S rRNA gene copies in the genome do not differ from each other, and do not differ from the previously published 16S rRNA gene sequence (AM943630), which contains two ambiguous base calls.

A representative genomic 16S rRNA gene sequence of *P. caeruleus* 13^T^ was compared using NCBI BLAST [25,26] under default settings (e.g., considering only the high-scoring segment pairs (HSPs) from the best 250 hits) with the most recent release of the Greengenes database [27] and the relative frequencies of taxa and keywords (reduced to their stem [28]) were determined, weighted by BLAST scores [Table 1]. The most frequently occurring genera were *Phaeobacter* (38.5%), *Ruegeria* (18.6%), *Roseobacter* (15.0%), *Silicibacter* (11.9%) and *Leisingera* (5.5%) (74 hits in total). Regarding the single hit to sequences from members of the species, the average identity within HSPs was 100.0%, whereas the average coverage by HSPs was 96.9%. Regarding the nine hits to sequences from other members of the genus, the average identity within HSPs was 97.6%, whereas the average coverage by HSPs was 99.5%. Among all other species, the one yielding the highest score was *Phaeobacter gallaeciensis* (AY881240), which corresponded to an identity of 98.3% and an HSP coverage of 99.3%. (Note that the Greengenes database uses the INSDC (= EMBL/NCBI/DDBJ) annotation, which is not an authoritative source for nomenclature or classification.) The highest-scoring environmental sequence was EF573869 (Greengenes short name 'site S25 near Coco's Island marine clone S25 213'), which showed an identity of 98.8% and an HSP coverage of 99.9%. The most frequently occurring keywords within the labels of all environmental samples which yielded hits were 'coral' (6.8%), 'caribbean' (5.8%), 'faveolata' (5.5%), 'chang' (5.4%) and 'disease-induc, montastraea, plagu, white' (5.2%) (169 hits in total). Environmental samples which yielded hits of a higher score than the highest scoring species were not found, indicating that the species is rarely found in environmental samples.

**Table 1:** Classification and general features of *P. caeruleus* DSM 24564^T^ according to the MIGS recommendations [29].

MIGS ID	Property	Term	Evidence code
		Domain *Bacteria*	TAS [30]
		Phylum *Proteobacteria*	TAS [31]
		Class *Alphaproteobacteria*	TAS [32,33]
	Current classification	Order *Rhodobacterales*	TAS [33,34]
		Family *Rhodobacteraceae*	TAS [34,35]
		Genus *Phaeobacter*	TAS [14,36]
		Species *Phaeobacter caeruleus*	TAS [1]
MIGS-7	Subspecific genetic lineage (strain)	13^T^	TAS [1]
MIGS-12	Reference for biomaterial	Vandecandelaere *et al*.	TAS [1]
	Gram stain	Gram-negative	TAS [1]
	Cell shape	Rod-shaped	TAS [1]
	Motility	Motile	NAS
	Sporulation	Not reported	
MIGS-6.1	Temperature range	4-45 °C	TAS [1]
MIGS-6.1	Optimum temperature	20°C	IDA
MIGS-6.3	Salinity	NaCl 2-5% (optimal, 3-4%)	TAS [1]
MIGS-22	Relationship to oxygen	Aerobe	TAS [1]
	Carbon source	Amino acid (tyrosine), DNA	TAS [1]
	Energy metabolism	Not reported	
MIGS-6	Habitat	Marine	TAS [1]
MIGS-6.2	pH	pH 6.0–9.0 (optimal, pH 6.5-8.0)	TAS [1]
MIGS-15	Biotic relationship	Biofilm	TAS [1]
MIGS-14	Known pathogenicity	Not reported	
MIGS-16	Specific host	Not reported	
MIGS-18	Health status of host	Not reported	
	Biosafety level	1	TAS [37]
MIGS-19	Trophic level	Not reported	
MIGS-23	Isolation	biofilm on stainless steel electrode	TAS [1]
MIGS-4	Geographic location	Italy, Genoa, harbor	TAS [1]
MIGS-5	Time of sample collection	before 2009	NAS
MIGS-4.1	Latitude	44.37	TAS [1]
MIGS-4.2	Longitude	8.94	TAS [1]
MIGS-4.3	Depth	Not reported	
MIGS-4.4	Altitude	Not reported	

Evidence codes - IDA: Inferred from Direct Assay; TAS: Traceable Author Statement (i.e., a direct report exists in the literature); NAS: Non-traceable Author Statement (i.e., not directly observed for the living, isolated sample, but based on a generally accepted property for the species, or anecdotal evidence). Evidence codes are from the Gene Ontology project [38].

### Morphology and physiology

**Figure 2:**
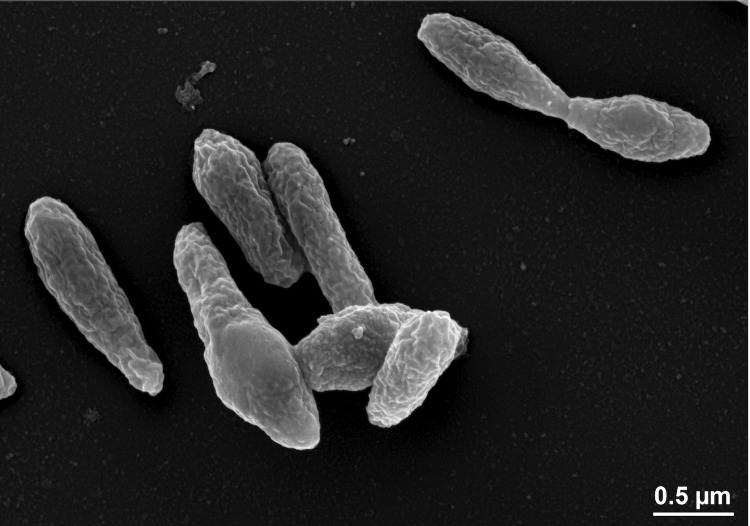
Scanning electron micrograph of *P. caeruleus* DSM 24564^T^

*P. caeruleus* 13^T^ cells are Gram-negative rods with a cell size of 0.9-1.8 µm (Figure 2). Bundles of polar flagella and inclusion bodies were observed by transmission electron microscopy (not visible in Figure 2). On marine agar the cells grow in round colonies with a surface of dark and bright blue circles, which becomes darker with incubation time [1].

The utilization of carbon compounds by *P. caeruleus* DSM 24564^T^ grown at 20°C was also determined for this study using Generation-III microplates in an OmniLog phenotyping device (BIOLOG Inc., Hayward, CA, USA). The microplates were inoculated at 28°C with a cell suspension at a cell density of 95-96% turbidity and dye IF-A. Further additives included vitamines, micronutrient and sea-salt solutions. The exported measurement data were further analyzed with the opm package for R [39,69], using its functionality for statistically estimating parameters from the respiration curves such as the maximum height, and automatically translating these values into negative, ambiguous, and positive reactions. The strain was studied in two independent biological replicates, and reactions with a different behavior between the two repetitions were regarded as ambiguous. At 28°C, the strain reacted poorly, with positive reactions only for 1% NaCl, 4% NaCl, lithium chloride, propionic acid and sodium bromate. This might be due to the optimum reported growth temperature of 20°C, whereas the phenotypic measurements were examined at 28°C. The result is in accordance with our observation that after incubation for 24 h in marine broth 2216 medium (MB; BD Biosciences, Franklin Lakes, NJ) and shaken at 100 rpm, *P. caeruleus* DSM 24564^T^ shows visible growth at 20°C but not at 28°C. Note, however, that [1] reported at least some growth for temperatures up to 45°C.

### Chemotaxonomy

Major fatty acids of *P. caeruleus* 13^T^ are C_18:1ω7c_, C_16:0_, an unknown fatty acid with an equivalent chain-length value of 11.7999, C_10:0 3-OH_, C_16:0 2-OH_, C_12:0 3-OH_, 11-methyl C_18:1ω7c_ and C_18:0_. The remaining fatty acids were present only in minor fractions and less than 1% of the total [1].

## Genome sequencing and annotation

### Genome project history

This organism was selected for sequencing on the basis of the DOE Joint Genome Institute Community Sequencing Program 2010, CSP 441: “Whole genome type strain sequences of the genera *Phaeobacter* and *Leisingera* – a monophyletic group of physiologically highly diverse organisms”. The genome project is deposited in the Genomes On Line Database [40] and the complete genome sequence is deposited in GenBank. Sequencing, finishing and annotation were performed by the DOE Joint Genome Institute (JGI) using state of the art technology [41]. A summary of the project information is shown in Table 2.

**Table 2:** Genome sequencing project information

MIGS ID	Property	Term
MIGS-31	Finishing quality	Non-contiguous finished
MIGS-28	Libraries used	Two Illumina paired-end libraries (270 bp and 8 kb insert size)
MIGS-29	Sequencing platforms	Illumina GAii, 454 GS FLX Titanium, PacBio
MIGS-31.2	Sequencing coverage	287 × Illumina
MIGS-30	Assemblers	Allpaths version 38445, Velvet 1.1.05, phrap version SPS - 4.24
MIGS-32	Gene calling method	Prodigal 1.4, GenePRIMP
	INSDC ID	Pending
	GenBank Date of Release	Pending
	GOLD ID	Gi10861
	NCBI project ID	77971
	Database: IMG	2512047087
MIGS-13	Source material identifier	DSM 24564
	Project relevance	Tree of Life, carbon cycle, sulfur cycle, environmental

### Growth conditions and DNA isolation

A culture of *P. caeruleus* DSM 24564^T^ was grown in DSMZ medium 514 [42] at 20°C. Genomic DNA was isolated using Jetflex Genomic DNA Purification Kit (GENOMED 600100) following the standard protocol provided by the manufacturer, but modified by the use of additional 10 µl proteinase K and 40 min incubation time. DNA is available through the DNA Bank Network [43].

### Genome sequencing and assembly

The draft genome sequence generated using Illumina sequencing technology. For this genome, we constructed and sequenced an Illumina short-insert paired-end library with an average insert size of 270 bp which generated 5,484,184 reads and an Illumina long-insert paired-end library with an average insert size of 7,670 +/- 2,475 bp which generated 4,839,808 reads totaling 1,549 Mb of Illumina data (Feng Chen, unpublished). All general aspects of library construction and sequencing performed can be found at the JGI web site [44]. The initial draft assembly contained 54 contigs in 17 scaffolds. The initial draft data was assembled with Allpaths [45] and the consensus was computationally shredded into 10 kbp overlapping fake reads (shreds). The Illumina draft data was also assembled with Velvet [46], and the consensus sequences were computationally shredded into 1.5 kbp overlapping fake reads (shreds). The Illumina draft data was assembled again with Velvet using the shreds from the first Velvet assembly to guide the next assembly. The consensus from the second Velvet assembly was shredded into 1.5 kbp overlapping fake reads. The fake reads from the Allpaths assembly and both Velvet assemblies and a subset of the Illumina CLIP paired-end reads were assembled using parallel phrap (High Performance Software, LLC) [47]. Possible mis-assemblies were corrected with manual editing in Consed [47]. Gap closure was accomplished using repeat resolution software (Wei Gu, unpublished), and sequencing of bridging PCR fragments with PacBio (Cliff Han, unpublished) technologies. A total of 45 additional sequencing reactions were completed to close gaps and to raise the quality of the final sequence. The final assembly is based on 1,549 Mbp of Illumina draft data, which provides an average 287 × coverage of the genome.

## Genome annotation

Genes were identified using Prodigal [48] as part of the JGI genome annotation pipeline [49], followed by a round of manual curation using the JGI GenePrimp pipeline [50]. The predicted CDSs were translated and used to search the National Center for Biotechnology Information (NCBI) nonredundant database, UniProt, TIGR-Fam, Pfam, PRIAM, KEGG, COG, and InterPro databases. Additional gene prediction analysis and functional annotation was performed within the Integrated Microbial Genomes – Expert Review (IMG-ER) platform.

## Genome properties

The genome statistics are provided in Table 3 and Figure 3. The assembly of the genome sequence consists of the genome sequence consists of three large scaffolds for the chromosome (3,520,924 bp, 564,457 bp and 447,629 bp in length, respectively) and six plasmids with sizes of 21,535 bp to 270,810 bp and a total G+C content of 63.3%. Of the 5,335 genes predicted, 5,227 were protein-coding genes, and 108 RNAs; 81 pseudo genes were also identified. The majority of the protein-coding genes (73.2%) were assigned a putative function while the remaining ones were annotated as hypothetical proteins. The distribution of genes into COGs functional categories is presented in Table 4.

**Table 3:** Genome Statistics

**Attribute**	**Value**	**% of Total**
Genome size (bp)	5,344,419	100.00
DNA coding region (bp)	4,713,144	88.19
DNA G+C content (bp)	3,380,828	63.27
Number of replicons	7	
Extrachromosomal elements	6	
Total genes	5,335	100.00
RNA genes	108	2.02
rRNA operons	4	
tRNA genes	92	1.72
Protein-coding genes	5,227	97.98
Pseudo genes	81	1.52
Genes with function prediction	3,904	73.18
Genes in paralog clusters	1,423	26.67
Genes assigned to COGs	3,844	72.05
Genes assigned Pfam domains	4,091	76.68
Genes with signal peptides	1,786	33.48
Genes with transmembrane helices	1,047	19.63
CRISPR repeats	1	

**Figure 3a:**
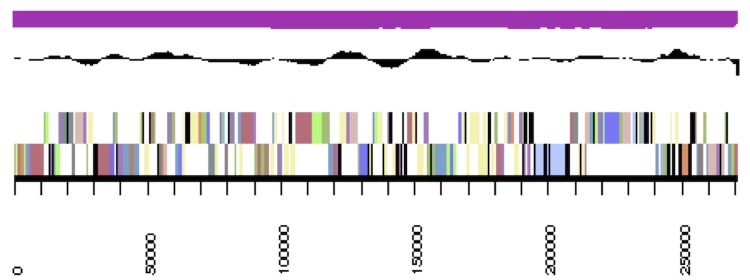
cCaer_A3521, DnaA. Graphical map of one of the scaffolds that constitute the chromosome. From bottom to top: Genes on forward strand (color by COG categories), genes on reverse strand (color by COG categories), RNA genes (tRNAs green, rRNAs red, other RNAs black), GC content, GC skew.

**Table 4:** Number of genes associated with the general COG functional categories

**Code**	**Value**	**%age**	**Description**
J	179	4.22	Translation, ribosomal structure and biogenesis
A	0	0	RNA processing and modification
K	346	8.16	Transcription
L	233	5.5	Replication, recombination and repair
B	3	0.07	Chromatin structure and dynamics
D	38	0.9	Cell cycle control, cell division, chromosome partitioning
Y	0	0	Nuclear structure
V	44	1.04	Defense mechanisms
T	224	5.29	Signal transduction mechanisms
M	194	4.58	Cell wall/membrane/envelope biogenesis
N	100	2.36	Cell motility
Z	2	0.05	Cytoskeleton
W	0	0	Extracellular structures
U	91	2.15	Intracellular trafficking, secretion, and vesicular transport
O	153	3.61	Posttranslational modification, protein turnover, chaperones
C	254	5.99	Energy production and conversion
G	175	4.13	Carbohydrate transport and metabolism
E	467	11.02	Amino acid transport and metabolism
F	103	2.43	Nucleotide transport and metabolism
H	194	4.58	Coenzyme transport and metabolism
I	176	4.15	Lipid transport and metabolism
P	192	4.53	Inorganic ion transport and metabolism
Q	143	3.37	Secondary metabolites biosynthesis, transport and catabolism
R	497	11.73	General function prediction only
S	430	10.15	Function unknown
-	1,491	27.95	Not in COGs

**Figure 3b:**
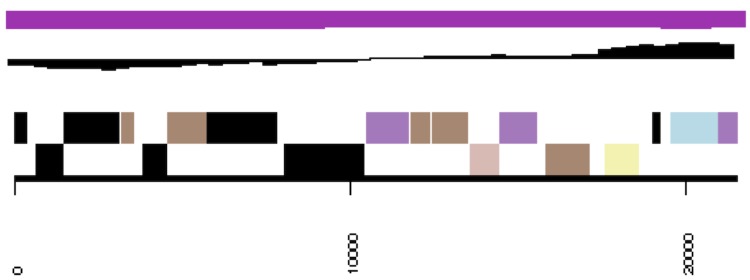
cCaer_B564, RepC-11. Graphical map of one of the scaffolds that constitute the chromosome. From bottom to top: Genes on forward strand (color by COG categories), genes on reverse strand (color by COG categories), RNA genes (tRNAs green, rRNAs red, other RNAs black), GC content, GC skew.

**Figure 3c:**
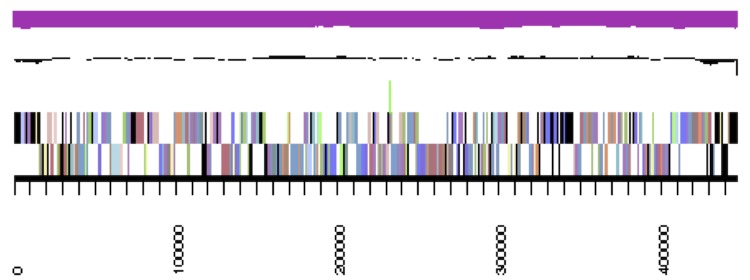
cCaer_C448. Graphical map of one of the scaffolds that constitute the chromosome. From bottom to top: Genes on forward strand (color by COG categories), genes on reverse strand (color by COG categories), RNA genes (tRNAs green, rRNAs red, other RNAs black), GC content, GC skew.

**Figure 3d:**
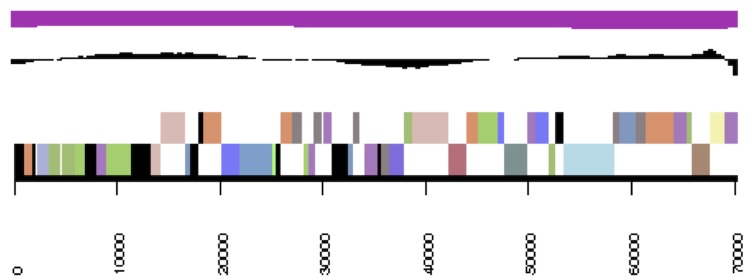
pCaer_A271, RepC-12. Graphical map of the plasmid. From bottom to top: Genes on forward strand (color by COG categories), genes on reverse strand (color by COG categories), RNA genes (tRNAs green, rRNAs red, other RNAs black), GC content, GC skew.

**Figure 3e:**
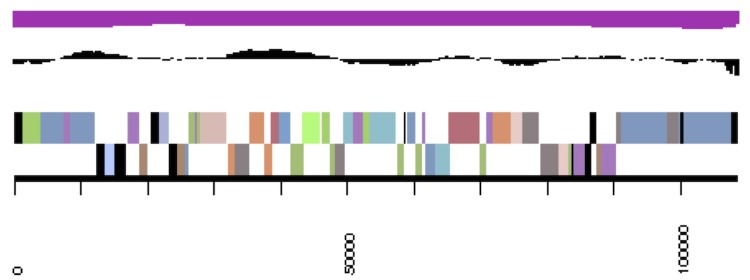
pCaer_B246, RepC-2. Graphical map of the plasmid. From bottom to top: Genes on forward strand (color by COG categories), genes on reverse strand (color by COG categories), RNA genes (tRNAs green, rRNAs red, other RNAs black), GC content, GC skew.

**Figure 3f:**
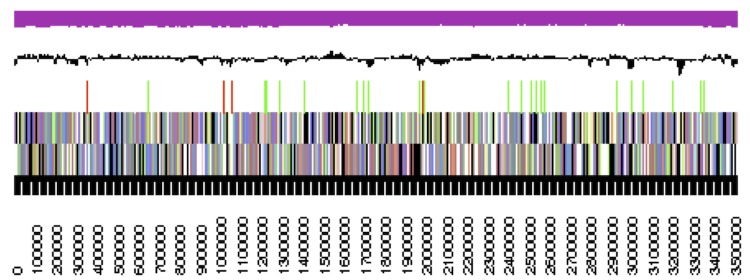
pCaer_C109, DnaA-like I. Graphical map of the plasmid. From bottom to top: Genes on forward strand (color by COG categories), genes on reverse strand (color by COG categories), RNA genes (tRNAs green, rRNAs red, other RNAs black), GC content, GC skew.

**Figure 3g:**
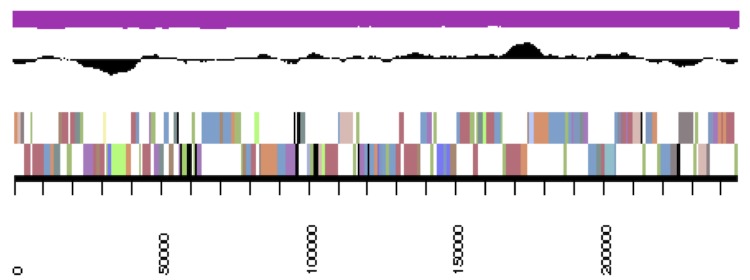
pCaer_D95, RepB-I. Graphical map of the plasmid. From bottom to top: Genes on forward strand (color by COG categories), genes on reverse strand (color by COG categories), RNA genes (tRNAs green, rRNAs red, other RNAs black), GC content, GC skew.

**Figure 3h:**
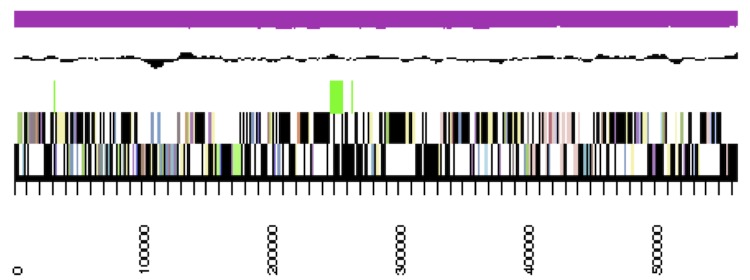
pCaer_E70, RepC-8. Graphical map of the plasmid. From bottom to top: Genes on forward strand (color by COG categories), genes on reverse strand (color by COG categories), RNA genes (tRNAs green, rRNAs red, other RNAs black), GC content, GC skew.

**Figure 3i:**
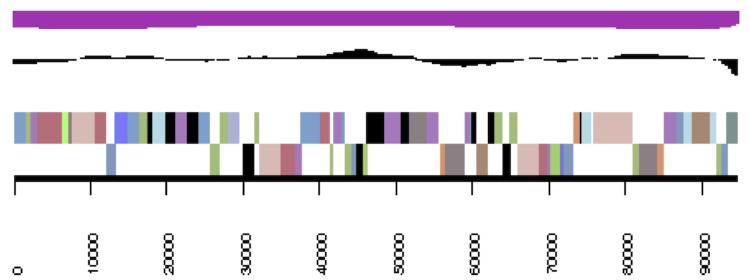
pCaer_F22, RepA-I. Graphical map of the plasmid. From bottom to top: Genes on forward strand (color by COG categories), genes on reverse strand (color by COG categories), RNA genes (tRNAs green, rRNAs red, other RNAs black), GC content, GC skew.

## Insights into the genome

Genome sequencing of *Phaeobacter caeruleus* DSM 24564^T^ resulted in nine scaffolds (contigs) with sizes between 22 kb and 3.5 MB (Table 5). The largest scaffold represents the chromosome as indicated by the presence of the typical replication initiation protein DnaA (Caer_2072) and the same affiliation can be assumed for scaffold 3 based on the absence of plasmid replication genes. The presence of more than 30 tRNA genes and CRISPRs (Clustered Regularly Interspaced Short Palindromic Repeats), which provide acquired resistance against viruses [52], on scaffold 2 is indicative for the chromosome. However, scaffold 2 does also contain a complete RepABC operon with genes for plasmid replication initiation (RepC-11; unpublished replication type) and partitioning (RepAB) as well as a perfect palindrome 5'-TTTACCG\CGGTAAA-3' that probably represents a functional cis-acting anchor for plasmid partitioning [53]. This peculiar distribution may either indicate the integration of a RepABC-11 type plasmid into the chromosome via recombination or an “outsourcing” of essential chromosomal genes to a plasmid that has recently been documented for the photosynthesis genes cluster of the *Roseobacter litoralis* [54].

**Table 5:** General genomic features of the chromosome and extrachromosomal replicons from *Phaeobacter caeruleus* strain DSM 24564^T^. ^*^circularity not experimentally validated; ^#^deduced from automatic annotation.

**Replicon**	**Scaffold**	**Replicase**	**Length** (bp)	**GC** (%)	**Topology**	**No. Genes^#^**
Chromosome	1	DnaA	3,520 924	64	linear*	3,453
Chromosome	2	RepC-11	564,457	60	linear*	657
Chromosome	3	-	447,629	64	linear*	468
pCaer_A271	4	RepC-12	270,810	60	linear*	277
pCaer_B246	5	RepC-2	245,600	65	linear*	212
pCaer_C109	6	DnaA-like I	108,530	65	linear*	89
pCaer_D95	7	RepB-I	94,628	67	linear*	91
pCaer_E70	8	RepC-8	70,306	67	linear*	66
pCaer_F22	9	RepA-I	21,535	66	linear*	22

The presence of plasmid replication modules on the remaining six fragments with sizes between 22 and 271 kb indicates that they all represent extrachromosomal elements, but their circularity has not been experimentally validated (Table 5). Three of the putative plasmids also contain RepABC-type operons representing the compatibility groups C-2, C-8 and C-12 [53]. The three remaining plasmids pCaer_C109, pCaer_D95 and pCaer_F22 represent DnaA-like I, RepB-I and RepA-I type plasmids, respectively [55,56]. The smallest plasmid pCaer_F22 contains the RepA-I type replicase, but a partitioning module is lacking. This distribution may correspond to a higher plasmid copy number within the cell thus assuring the replicon maintenance in the daughter cells after cell division.

The locus tags of all replicases, plasmid stability modules and the large *virB4* and *virD4* genes of type IV secretion systems are presented in Table 6. The plasmids pCaer_B246 and pCaer_C109 contain postsegregational killing systems (PSKs) consisting of a typical operon with two small genes encoding a stable toxin and an unstable antitoxin [57]. The largest plasmid pCaer_A271 contains a complete type IV secretion system including the *virB* operon for the formation of a transmembrane channel. The relaxase VirD2, which is required for the strand-specific DNA nicking at the origin of transfer (*oriT*), and the coupling protein VirD4 support the presence of functional conjugation system [58,59]. The DnaA-like I replicon pCaer_C109 contains a large type VI secretion system (T6SS) with a size of about 30 kb. The role of this export system that has been first described in the context of bacterial pathogenesis, but recent findings indicate a more general physiological role in defense against eukaryotic cells and other bacteria in the environment [60]. Homologous T6S systems are present on the DnaA-like I plasmids of *Leisingera aquimarina* DSM 24565^T^ (pAqui_F126) and *L. methylohalidivorans* DSM 14336^T^ (pMeth_A285) as well as the RepC-8 type plasmid of *Phaeobacter daeponensis* DSM23529^T^ (pDaep_A276).

**Table 6:** Integrated Microbial Genome (IMG) locus tags of *P. caeruleus* DSM 24564^T^ genes for the initiation of replication, toxin/antitoxin modules and two representatives of type IV secretion systems (T4SS) that are required for conjugation. The locus tags are accentuated in blue.

**Replicon**	**Replication Initiation**		**Plasmid Stability**		**Type IV Secretion**	
	Replicase	Locus Tag	Toxin	Antitoxin	VirB4	VirD4
Chromosome	DnaA	Caer_2072	-	-	-	-
Chromosome	RepC-11	Caer _5060	-	-	-	-
Chromosome	-	-	-	-	-	-
pCaer_A271	RepC-12	Caer _0252	-	-	Caer _0206	Caer _0215
pCaer_B246	RepC-8	Caer _4471	Caer _4419	Caer _4420	-	-
pCaer_C109	DnaA-like I	Caer _0297	Caer _0862	Caer _0863	-	-
pCaer_D95	RepB-I	Caer _5279				
pCaer_E70	RepC-2	Caer _0776				
pCaer_F22	RepA-I	Caer _0297				

Several strains affiliated with the *Roseobacter* clade show a high potential to produce secondary metabolites [51]. Pigmentation of cells is often related with secondary metabolite production [61]. We assume that the characteristic blue color of *P. caeruleus* is attributed to the production of the blue pigment indigoidine. In the closely related and blue-colored *Phaeobacter sp.* strain Y4I indigoidine is produced *via* a non-ribosomal peptide synthase (NRPS)-based biosynthetic pathway encoded by the gene cluster *igiBCDFE* [62]. In strain Y4I indigoidine production is correlated with pleiotrophic effects, such as motility, resistance to hydrogen peroxide, surface colonization and inhibition of *Vibrio fischeri*. A cluster analysis revealed that the *P. caeruleus* plasmid pCaer_B246 contains a homologous *igiBCDFE* gene cluster (Caer_4407 - Caer_4412). Thus it seems likely that *P. caeruleus* can also produce the antimicrobial secondary metabolite indigoidine *via* its NRPS cluster. Therefore, indigoidine could be the pigment responsible for the blue color and *P. caeruleus* could have inhibitory effects on other bacteria.

Mutants in either of the two LuxIR systems in *Phaeobacter sp.* strain Y4I are lacking the indigoidine production, therefore, quorum sensing seems to play a role in its biosynthesis [62]. A correlation between quorum sensing and pigmentation and antimicrobial effects is already known for members of the *Roseobacter* clade. The LuxIR-type quorum sensing system of *P. inhibens* DSM 17395 (originally deposited as *P. gallaeciensis* DSM 17395; Buddruhs *et al.*, unpublished) regulates N-acyl homoserine lactones production which co-occurs with the strains dark pigmentation and antibiotic activity [63]. The *P. caeruleus* DSM 24564^T^ chromosome cCaer_A3521 has a *luxIR* gene cluster (Caer_1365 - Caer_1371) which shows strong homology to the mentioned LuxIR-type cluster of *P. inhibens* DSM 17395 and strain Y4I, thus pigmentation and putative inhibitory effects could be regulated via quorum sensing. Besides these *luxIR* genes, five other *luxIR* clusters are encoded in the genome of strain DSM 24564^T^ which could play an important role in cell-cell signaling.

Recently siderophore production was shown for *P. inhibens* DSM 17395 [64]. Distinct siderophore transport systems such as an ABC-type enterobactin transport system, two ABC-type cobalamin/Fe3+-siderophores transport systems, two ABC-type Fe3+-siderophore transport systems, two ABC-type Fe3+-hydroxamate transport systems, a TonB-dependent siderophore receptor and a siderophore-interacting protein are encoded in the genome of *P. caeruleus* (Caer_4537, Caer_1186, Caer_4536, Caer_1187, Caer_4538, Caer_1188, Caer_4539, Caer_4530, Caer_4535). But only one gene, encoding a phosphopantetheinyl transferase component of a siderophore synthetase, is associated with siderophore biosynthesis (Caer_3105). As it was isolated from a biofilm and a siderophore-transport associated genes were present, we presume that *P. caeruleus* DSM 24564^T^ is utilizing siderophores, which are produced by other ambient bacteria [65].

The phylogenetic tree of the 16S rRNA gene analysis (Figure 1) with intermingled *Phaeobacter* and *Leisingera* species indicates that the classification of *P. caeruleus* DSM 24564^T^ might need to be reconsidered. Hence, we conducted a preliminary phylogenomic analysis using GGDC [66-68] and the draft genomes of the type strains of the other *Leisingera* and *Phaeobacter* species. The results shown in Table 7 indicate that the DNA-DNA hybridization (DDH) similarities calculated in silico for *P. caeruleus* DSM 24564^T^ compared to other *Phaeobacter* species are, in general, not higher than those to *Leisingera* species. Although, the highest value by far was obtained for *P. daeponensis*, it was immediately followed by *L. aquimarina* and *L. methylohalidivorans*, which is in accordance with Figure 1.

**Table 7:** DDH similarities between *P. caeruleus* DSM 24564^T^ and the other *Phaeobacter* and *Leisingera* species (with genome-sequenced type strains) calculated in silico with the GGDC server version 2.0 [66]*.

**Reference species**	**formula 1**	**formula 2**	**formula 3**
*L. aquimarina* (2516653083)	45.90±3.41	28.40±2.44	40.60±3.01
*L. methylohalidivorans* (2512564009)	45.80±3.41	27.00±2.42	39.90±3.0
*L. nanhaiensis* (2512047090)	14.50±3.11	19.40±2.29	14.60±2.65
*P. arcticus* (2516653081)(2512047087)	16.90±3.26	20.40±2.32	16.70±2.76
*P. daeponensis* (2516493020)	62.50±3.67	40.30±2.51	57.80±3.18
*P. gallaeciensis* (AOQA01000000)	17.90±3.31	21.40±2.34	17.70±2.80
*P. inhibens* (2516653078)	18.20±3.32	21.50±2.34	17.90±2.81

*The standard deviations indicate the inherent uncertainty in estimating DDH values from intergenomic distances based on models derived from empirical test data sets (which are always limited in size); see [66] for details. The distance formulas are explained in [67]. The numbers in parentheses are IMG object IDs (GenBank accession number in the case of *P. gallaeciensis*) identifying the underlying genome sequences.
